# Differential Diagnosis and Treatment of Restless Legs Syndrome: A Literature Review

**DOI:** 10.7759/cureus.3297

**Published:** 2018-09-13

**Authors:** Vishal Kwatra, Muhammad Adnan Khan, Syed A Quadri, Trevor S Cook

**Affiliations:** 1 Internal Medicine, St. Vincent Charity Medical Center, Cleveland, USA; 2 Neurosciences & Neurology, California Institute of Neurosciences, Thousand Oaks, USA; 3 Neurosurgery, California Institute of Neurosciences, Thousand Oaks, USA; 4 Emergency, Palacios Community Hospital, Palacios, USA

**Keywords:** restless legs syndrome, rls, willis-ekbom disease, wed, augmentation, dopamine agonist, dopamine agonist

## Abstract

Restless legs syndrome (RLS) is a chronic neurological disorder affecting a growing number of people. Patients describe an irresistible urge to move their lower limbs in times of immobility. Due to its vague symptoms and similarity to other disorders, it has become increasingly difficult for primary care clinicians to properly diagnose and manage RLS. As a result, patients with RLS are routinely misdiagnosed and continue their lives without proper management. This literature review examines the current understanding of the disorder, provides key points to assist clinicians in differentiating RLS from similar disorders, and explores recently updated evidence-based guidelines for the effective management of RLS.

## Introduction and background

Restless legs syndrome (RLS)/Willis-Ekbom disease (WED), is a neurological sensory-motor disorder that often disturbs sleep [1]. It is characterized by patients as an uncomfortable urge to move their legs in conditions of immobility [1]. Often, the urge to move is accompanied by an unpleasant sensation. Both symptoms are transiently relieved through movement but once the patient ceases activity, the symptoms may return [2]. In very severe cases of RLS, prolonged periods of activity may provide minimal to no relief of symptoms [3]. Therefore, without proper management of RLS, it is common for patients to live their lives experiencing functional impairment [4]. Numerous studies have highlighted primary-care physicians’ lack of awareness to adequately diagnose the disorder [5-7]. As a result, this leads to a patients’ symptoms being attributed to other medical conditions, such as peripheral neuropathy, leg cramps, and anxiety [8]. At times, patients are simply referred to other specialists for further investigation. The exact prevalence differs depending on the specific ethnic population sampled and the diagnostic criteria used to define RLS. Ohayon, O'Hara, and Vitiello reviewed 47 epidemiology studies of RLS published between 1994 and 2010 [9]. Overall, the prevalence of RLS ranged from 5% to 10% of adults in North America and Europe [9]. Of the 47 studies, 10 applied a differential diagnosis which yielded a lower prevalence of 1.9% to 4.6% [9]. In the last decade, the method of evaluation and management of RLS has gone through significant changes. This paper serves to update primary care clinicians’ familiarity with RLS by detailing its clinical features, diagnostic criteria, differential diagnosis, and evidence-based recommendations for effective management of RLS.

## Review

The diagnostic criterion for RLS has continually evolved from the broad diagnostic criteria released by Ekbom in the 1960s [10] to the first formal RLS description established in 1979 [11], which was scrutinized for not being reflective of the clinical data [3]. Due to the perceived inadequacy of current guidelines, an international interdisciplinary collaboration between the National Institute of Health (NIH) and the International Restless Legs Syndrome Study Group (IRLSSG) formed and released the NIH/IRLSSG criteria in 2003 [12]. Major changes were included in the updated criteria such as replacing the confusing 'motor restlessness' term with 'urge to move' description, establishing a new diagnostic criteria specifically for children and the cognitively impaired elderly [12]. The updated criteria quickly became the new diagnostic standard, but this evaluation fell short of considering a differential diagnosis [13]. Studies that were done post-2003, incorporating these criteria, suffered from higher false positives and inflated prevalence markers [13]. As a result, the NIH/IRLSSG criteria were revised, and the updated 2012 version (Table 1) included an additional fifth criterion stressing upon differential diagnosis, thereby lowering the false positives and improving specificity [3]. The diagnosis of RLS rests on the subjective description of the patient's symptoms [3]. To be formally diagnosed with RLS, all five IRLSSG diagnostic criteria must be satisfied [3].

**Table 1:** IRLSSG diagnostic criteria IRLSSG: International Restless Legs Syndrome Study Group

IRLSSG diagnostic criteria:
An urge to move the legs, usually accompanied or caused by uncomfortable and unpleasant sensations in the legs. Sometimes the urge to move is present without the uncomfortable sensations, and sometimes the arms or other body parts are involved in addition to the legs.
The urge to move or unpleasant sensations begin or worsen during periods of rest or inactivity such as lying or sitting.
The urge to move or unpleasant sensations are partially or totally relieved by movement, such as walking or stretching, at least as long as the activity continues.
The urge to move or unpleasant sensations are worse in the evening or night than during the day or only occur in the evening or night. When symptoms are severe, the worsening at night may not be noticeable but must have been previously present.
Symptoms are not solely accounted for by another medical or behavioral condition, such as leg cramps or habitual foot tapping.
Supportive criteria:
• A family history of RLS.
• A positive response to dopaminergic drugs.
• Periodic limb movements during wakefulness or sleep as assessed with polysomnography or leg activity devices.

Differential diagnosis

Thanks to the fifth criterion, a greater emphasis is now being placed to differentiate other conditions that mimic RLS. Certain conditions can occasionally possess traits that meet four of the five requirements, such as leg cramps and positional leg discomfort. Nocturnal leg cramps are presented as unilateral, painful involuntary muscle contractions, [14] which also share a circadian pattern of commonly occurring at rest [15]. However, they can enter into brief spasms which, unlike RLS, can be relieved (at least temporarily) by dorsiflexion of the foot [16] as well as stretching [15]. Patients with positional leg discomfort experience pain in a small localized area [16]. The pain is brought on by prolonged sitting and lacks a circadian pattern [17]. RLS symptoms differ in both conditions by involving larger parts (i.e., thighs and/or calves) and exhibiting prolonged symptoms that can only be relieved by walking or other activities [16].

Patients that complain of sensory discomfort when standing but improve with movement may be describing a venous disorder [17]. Venous disorders do not follow a circadian pattern nor are they improved by dopaminergic therapy, but skin alterations may be observed [17]. Similarly, skin alterations may also be noted in vascular or neurogenic claudication [17]. Vascular intermittent claudication is a consequence of inadequate delivery of oxygenated blood flow to the peripheral tissues [18]. It is a frequent cause of leg pain and discomfort [18]. In contrast to RLS, intermittent claudication occurs during activity, is relieved by rest, and lacks the urge to move [19].

Akathisia is a condition that is commonly associated with neuroleptic medications and is characterized by a general feeling of inner restlessness and an inability to sit still during the day [20]. This inner restlessness can affect the whole body and can result in varying motor manifestations such as a difficulty in crossing and uncrossing legs, constantly changing sitting positions, or rocking the whole body [19]. In contrast, RLS has a localized area of paresthesia in the limbs, is most active during evening/night affecting sleep, and has no history of exposure to neuroleptic medications or associated extrapyramidal symptoms [8].

Polyneuropathy involves painful burning sensations in the upper and lower extremities [21]. It is also associated with sensory paraesthesia and sensitivity to the touch of the skin [21]. The important distinction with polyneuropathy involves the lack of motor restlessness, circadian pattern [21], and the improvement of symptoms through movement or dopaminergic therapy [8]. Lumbosacral radiculopathy also differs from RLS due to its lower back pain, and often an asymmetrical sensory paresthesia [17]. It is also possible to have peripheral neuropathy/radiculopathy with RLS [8].

Parkinson's disease (PD) can sometimes present with RLS. Although both conditions are improved with dopaminergic therapy, their pathologies are distinct [22]. It's important to note that PD can also mimic RLS symptoms by presenting with akathisia and 'leg motor restlessness' [20]. 'Leg motor restlessness' is a diagnosis given to PD patients who are experiencing an urge to move their legs, but does not fulfill the essential criteria for RLS [20]. The exact prevalence of RLS in PD patients has been variable, ranging from 0-52% between studies [23]. This may be due to the prevalence of studies neglecting to differentiate the stage of PD, not distinguishing between drug-naive patients and patients receiving dopaminergic therapy, or accounting those with leg motor restlessness [22]. As such, it’s imperative to utilize the IRLSSG diagnostic criteria to properly distinguish RLS from other pathologies.

Treatment

Although RLS can be a debilitating disorder, it is a treatable condition that is responsive to non-pharmaceutical and pharmaceutical therapy. The restless leg foundation recommends that clinicians base their therapy on disease severity and patient's comorbidities [24]. Patients’ symptoms can be grouped into three categories: intermittent (periodic treatment), chronic persistent RLS (daily therapy), and refractory RLS (dopamine agonists ineffective/intolerable adverse effects/augmentation effect) [24]. Basing treatment on the number of episodes per week can be limited by the severity of each specific episode.

Iron Therapy

Currently, there is an extensive volume of clinical studies suggesting that brain iron deficiency plays a role in developing RLS [25]. This association was first reported by Dr. Nordlander who documented 10 of his iron anemic patients with RLS [26]. In this study, the patients’ RLS symptoms are resolved with a series of intravenous iron therapy [26].

A prevalence study involving 343 patients with iron-deficient anemia (IDA) found that almost a quarter were diagnosed with the symptoms of RLS [27]. This study was clinically significant as it quantifies that these IDA patients were nine times more likely to have RLS than the general population [27]. 

Clinicians are encouraged to evaluate the iron status of all patients presenting with signs and symptoms of RLS [24]. Patients with serum ferritin below 75ng/L have demonstrated an improvement of symptoms with iron supplementation [24-25]. The general consensus is to prescribe 325mg of oral ferrous sulfate to be taken daily along with 100mg to 200mg of vitamin C to increase iron absorption [24-25].

In recent years, there has been a rise in the clinical trials treating RLS through oral and intravenous (IV) methods [25]. IV iron supplementation is known to quickly replenish iron stores and requires fewer administrations than oral therapy, yet suffers from being less convenient [25]. IV formulations are usually considered when oral therapy cannot be tolerated by the patient if the iron formulation cannot be orally absorbed, or oral iron therapy cannot keep pace with rapid iron loss (eg. acute blood loss) [24-25].

Non-pharmacological Therapy

Non-pharmaceutical therapy is often recommended in all categories of treatment. However, the application of these following therapies is based on an insufficient number of randomized controlled trials (RTC) [28]. Common strategies that are recommended to patients are abstinence from alcohol and caffeinated drinks, proper sleep hygiene, and pneumatic compression devices [28]. A few clinical trials have demonstrated a positive benefit by completing moderate exercise. A 12-week trial involving 23 participants engaged in aerobic and lower-body resistance training resulted in improvement of RLS symptoms [29]. Furthermore, a meta-analysis involving four clinical studies concluded a reduction of RLS symptom severity through moderate exercise [30].

In May 2014, an alternative therapy vibration device had been approved by the US Food and Drug Administration (FDA) for patients with RLS. A non-MEDLINE-indexed journal published the results of two randomized studies which investigated the vibration pads against sham treatments. Both studies concluded RLS symptom improvement [31]. However, when the results were analyzed using the International Restless Legs Syndrome Study Group (IRLSSG) scores or RLS quality of life scores, they revealed no substantial benefit. Based on the current data that is available, we cannot find significant evidence that supports the noteworthy benefits of using vibration pads to alleviate RLS symptoms.

Intermittent Symptoms

Although symptoms in this category occur less than twice a week, it is however of still enough severity to justify treatment [24]. Mild to intermittent symptoms can sometimes be controlled with non-pharmacological therapies alone [24]. If symptoms remain uncontrolled and disruptive, then the addition of pharmaceutical medication is clinically recommended intermittently [24]. Typical medications incorporated include carbidopa/levodopa, dopamine agonists, low-potency opioid analgesics (codeine or tramadol) or sedative hypnotics (temazepam or zolpidem) [28]. It is important to note that the utilization of a low dose-controlled release of carbidopa/levodopa can still pose a risk for augmentation or rebound complications.

Chronic Persistent RLS

The symptoms of chronic, persistent RLS are characterized as moderate to severe, occurring at least twice a week, thereby requiring treatment [24]. Before discussing pharmaceutical therapy, clinicians should clarify with their patient that the objective is not to completely cure their symptoms but to ensure they do not impede with their quality of life [32]. The initial treatment usually involves either dopamine agonists or α2δ calcium channel ligand drugs [28, 33]. In the past, choosing between the two groups of medications was mainly based on the specific patient profile, however, updated international guidelines now recommend α2δ calcium channel ligand medications as the initial drug of choice [32, 34-36]. This decision is based on the significant limitations of dopaminergic agonists. Through the long-term clinical trials using dopamine agonists, patients have reported a progressive loss of therapeutic response [34] and become at risk of developing augmentation [1, 32, 34, 37-39]. Other pharmaceutical options include low-to-medium potency opioids [34].

Utilization of α2δ calcium channel ligand medication should be given further preference over dopamine agonists, particularly in cases where the patient suffers from chronic pain, anxiety, insomnia, or impulse control disorders (ICD) [1]. Conversely, dopamine agonists can be considered in circumstances where the patient presents with severe symptoms of depression, obesity/metabolic syndrome, a risk of falls, or cognitive impairment, as these conditions may be exacerbated with α2δ calcium channel ligand integration [1]. Currently, there are three α2δ calcium channel ligands used to treat RLS: gabapentin, gabapentin enacarbil, and pregabalin (Table 2) [32].

**Table 2:** Recommended dosage of α2δ calcium channel ligands

α2δ calcium channel ligand	Initial dose	Effective dose range
Gabapentin enacarbil	600 mg	600 – 1200 mg
Pregabalin	75 mg	150 – 450 mg
Gabapentin	300 mg	900 – 1200 mg

Gabapentin has preferential treatment when the patient presents with painful RLS or RLS-associated polyneuropathy [40-41]. Since gabapentin is known to produce peripheral edema, dizziness, and somnolence [42] a 300mg dose can be given two to three hours before bedtime [34]. Start with a small dose and increase the dosage weekly until symptoms improve or the maximum dose of 900mg to 2000mg has been reached. Caution should be practiced with prescribing gabapentin in those with renal impairment [42].

A large-scale, 52-week, randomized, double-blind trial has assessed the efficacy between pregabalin, pramipexole 0.25mg, pramipexol 0.5mg, and placebo (three months of placebo followed by 40 weeks of a randomly assigned active treatment) [35]. With 719 participants, the study found that pregabalin had greater efficacy than 0.25mg of pramipexol [35]. Furthermore, the augmentation rates were significantly greater in the pramipexol 0.25mg and 0.5mg group (6.6% and 9.0% respectively) than pregabalin group (1.7%) [35]. Pregabalin has been known to increase the risk of suicidal ideation, dizziness [34-35, 42], fatigue, and weight gain [34-35]. Despite these risks, pregabalin has clinically demonstrated to improve treatment outcomes and provides a significantly lower augmentation rate when compared to dopamine agonists.

Gabapentin enacarbil is a slow-releasing prodrug of gabapentin. Of the α2δ calcium channel ligands, only gabapentin enacarbil is an FDA-approved treatment for RLS. Numerous RCT studies have demonstrated its clinical efficacy in reducing RLS symptoms [43-45]. Currently, there are an insufficient number of long-term studies that investigate its efficacy or adverse effects beyond a 12-week period. One 52-week study found that 80% of the participants reported mild to moderate adverse effects and a withdraw rate of 20% [46]. Common adverse effects include dizziness and somnolence which were found to be dose dependent [42].

Dopaminergic medications are one of the most widely studied and used therapies for RLS. A meta-analysis that reviewed 29 RCT studies using dopamine agonists found a reduction of symptoms greater than 50% (as defined by international restless syndrome scale) when compared with placebo (61% vs 41%) [33].

The FDA-approved three dopamine agonist medications for the treatment of RLS: ropinirole, pramipexole, and rotigotine [24]. It is typically recommended to begin therapy using the lowest dose (Table 3) and instruct the patient to take the medication two hours before the onset of RLS symptoms to allow the medication time to take effect [24, 32]. To provide relief, the dosage may cautiously be increased by 1mg at the end of the week but never exceed the recommended dosage [32]. Common adverse effects include nausea, headache, dizziness, and vomiting which typically resolve within two weeks [47].

**Table 3:** Dopamine agonist recommended dosage

Dopamine agonist	Initial dose	Maximum dose
Pramipexole	0.125 mg/day	0.75 mg/day
Ropinirole	0.25 mg/day	4 mg/day
Rotigotine	1 mg/day	3 mg/day

RLS patients receiving dopamine agonists have an increased risk of developing augmentation or impulse control disorders (ICD) such as pathologic gambling, hypersexuality, compulsive shopping, and compulsive eating [28]. Initially, drug-induced augmentation was mostly attributed to the use of levodopa [48]. However, the phenomenon has also been reported in long-term trials of dopamine agonists. In fact, within a year of short dopamine agonist use, the incidence rate of augmentation was 7% to 8% [1]. Several retrospective studies have reported that the incidence of augmentation rates jump to 50% after a 10-year period [39]. Because augmentation worsens over time and RLS is a chronic disease requiring ongoing treatment, prescribing α2δ calcium channel ligands have slowly gained favor over dopamine agonists.

Augmentation presents as an increase in RLS symptom severity after a dosage of medication [38]. It can sometimes be confused for drug tolerance where the therapeutic benefit of the medication diminishes over time. Augmentation shares this quality, but also involves the worsening of symptoms beyond the level of severity at the time of initial treatment [37]. This phenomenon can elicit early daytime RLS symptoms, an increased intensity of symptoms, and distribution of symptoms to other previously unaffected (eg. arms) [38, 49].

Evidence-based guidelines for the management of augmentation were established by the IRLSSG in conjunction with the European Restless Legs Syndrome Study Group (EURLSSG) and the Restless Legs Syndrome Foundation (RLSF). It is recommended to first investigate any aggravating factors. Such factors can include ruling out low serum ferritin, specific medications known to exacerbate RLS (eg. recent opioid discontinuation or anti-depressants), and lifestyle modifications [32, 38]. Next would be to determine the severity of the augmentation symptoms and group them between mild and severe.

Under mild augmentation, the clinician can keep the original dosage, but splits it between a nighttime and daytime dose, or starts the dose before symptoms begin [32]. A different approach can be to increase the dopaminergic drug dosage [32]. However, careful attention should be made not to exceed the recommended total daily dosage. Alternatively, the clinician can completely switch out the original dopaminergic medication with a long-acting dopamine agonist (eg. Rotigotine) or α2δ calcium channel ligands [37-38].

For severe augmentation symptoms, the guidelines suggest three options. One would be to completely switch medication to a long-acting dopamine agonist [32]. The second option involves adding an α2δ calcium channel ligand and gradually reducing the original dopamine agonist [32]. Although the goal is to abolish the dopaminergic medication while on an α2δ calcium channel ligand, the patient needs to be warned that this approach can temporarily worsen their symptoms [38]. If attempting to eliminate the dopamine drug fails, then adding a low-dose dopamine agonist with an α2δ calcium channel ligand can be continued [37]. The third and last option includes a gradual weaning off the dopamine agonist followed by a 10-day washout period [32]. This approach can lead to extremely severe RLS symptoms that may require a low-dose dopamine agonist to provide some relief [37].

Refractory RLS

When monotherapy proves ineffective due to tolerance, augmentation, or adverse effects, physicians should first consider potential factors affecting therapy such as the use of drugs potentiating RLS, a change in sleep habits, or other pathologies disturbing sleep [24]. A common protocol includes combining primary therapy with a different class of drug (eg. an α2δ calcium channel ligand for patients treated with dopamine agonists or vice versa) [3, 34]. Since both provide therapeutic benefit through different pharmacological mechanisms of action, the combination therapy may produce superior symptom control [34]. Furthermore, iron levels of the patients can also be rechecked and treated with iron therapy if low iron levels are discovered [3].

Long-term Therapy

To date, a majority of the evidence-based treatment guidelines were established from sourcing studies that were conducted for no more than four months in length. This limitation is significant, as RLS is a lifelong disease and potential adverse effects from long-term drug therapy were not investigated. Levodopa’s drug effectiveness can last up to two years for up to 40% of patients, while the effectiveness of non-ergot dopamine agonists like pramipexole and rotigotine lasts one and five years, respectively [1]. Concerning α2δ calcium channel ligand medications, pregabalin, and gabapentin enacarbil have proven effective for one-year treatments [1]. Other medications lack sufficient evidence to conclude their long-term effectiveness, such as gabapentin and many opioid medications. Due to the lack of evidence, it is clear that further research is warranted in studying the long-term pharmaceutical therapy of RLS. Some drugs like pergolide and cabergoline have been completely discontinued in the treatment of RLS due to reports of cardiac valve fibrosis [1, 34].

## Conclusions

Although major advancements have occurred in the etiology, diagnosis, and management of RLS in the last decade, patients are continually suffering from misdiagnosis and multiple unnecessary referrals. Through advancements in the diagnostic criteria, treatment strategies, and updated guidelines, primary care physicians have more resources than ever to diagnose and effectively manage RLS patients. Future advancement in drug therapy guidelines rests on continued research in investigating pharmaceutical long-term therapy outcomes.
